# Indirect targeting of IGF receptor signaling *in vivo* by substrate-selective inhibition of PAPP-A proteolytic activity

**DOI:** 10.18632/oncotarget.1629

**Published:** 2014-01-17

**Authors:** Jakob H. Mikkelsen, Zachary T. Resch, Bhanu Kalra, Gopal Savjani, Ajay Kumar, Cheryl A. Conover, Claus Oxvig

**Affiliations:** ^1^ Department of Molecular Biology and Genetics, University of Aarhus, DK-8000 Aarhus C, Denmark; ^2^ Endocrine Research Unit, Division of Endocrinology, Mayo Clinic, Minnesota, MN, USA; ^3^ Ansh Labs, Webster, TX, USA

**Keywords:** insulin-like growth factor receptor, pregnancy-associated plasma protein-A, proteolytic inhibition, therapeutic antibody, xenograft mouse model

## Abstract

The insulin-like growth factor (IGF) signaling pathway is involved in certain human cancers, and the feasibility of directly targeting the IGF receptor has been actively investigated. However, recent evidence from clinical trials suggests that this approach can be problematic. We have developed an alternative strategy to indirectly inhibit the IGF signaling by targeting the metalloproteinase, pregnancy-associated plasma protein-A (PAPP-A). PAPP-A associated with the cell surface cleaves IGF binding protein-4 (IGFBP-4), when IGF is bound to IGFBP-4, and thereby increases IGF bioavailability for receptor activation in an autocrine/paracrine manner. We hypothesized that inhibition of PAPP-A would suppress excessive local IGF signaling in tissues where this is caused by increased PAPP-A proteolytic activity. To test this hypothesis, we developed an inhibitory monoclonal antibody, mAb 1/41, which targets a unique substrate-binding exosite of PAPP-A. This inhibitor selectively and specifically inhibits proteolytic cleavage of IGFBP-4 with an inhibitory constant (Ki) of 135 pM. In addition, it inhibited intracellular signaling of the IGF receptor (AKT phosphorylation) in monolayers of A549 cells, an IGF-responsive lung cancer-derived cell line found to express high levels of PAPP-A. We further showed that mAb 1/41 is effective towards PAPP-A bound to cell surfaces, and that it is capable of inhibiting PAPP-A activity in vivo. Using a murine xenograft model of A549 cells, we demonstrated that mAb 1/41 administered intraperitoneally significantly inhibited tumor growth. Analysis of xenograft tumor tissue recovered from treated mice showed penetration of mAb 1/41, reduced IGFBP-4 proteolysis, and reduced AKT phosphorylation. Our study provides proof of concept that IGF signaling can be selectively reduced by targeting a regulatory proteinase that functions extracellularly, upstream of the IGF receptor. PAPP-A targeting thus represents an alternative therapeutic strategy for inhibiting IGF receptor signaling.

## INTRODUCTION

The insulin-like growth factor receptor type I (IGF-IR) and its native ligands, IGF-I and IGF-II, play crucial regulatory roles in many cellular processes, particularly during normal development and growth [1, 2]. In addition, dysregulated IGF-IR signaling has been linked to various diseases, especially cancer [3-6]. In fact, the relationship between the IGF system and cancer has been extensively studied for decades. It includes early evidence that cancer cell lines secrete IGF-I [7], express the IGF-IR on their surface [8], and that IGF-IR is required for embryonic cells to be transformed by oncogenes [9]. Furthermore, numerous epidemiological studies have linked the circulating level of IGF-I to cancer risk [5, 10].

For these reasons it is not surprising that major efforts have focused on targeting IGF-IR signaling in cancer therapy. Several different strategies have been developed, including the use of 1) anti-ligand antibodies to reduce the concentration of bioactive ligand capable of stimulating the receptor, 2) anti-receptor antibodies, which trigger receptor internalization and block binding of active IGF-I or -II, and 3) small molecule inhibitors of the intracellular receptor tyrosine kinase domain [11, 12]. Although all of these strategies have made the transition into clinical trials, each of them is connected with potential problems, and the outcomes have been less fruitful than anticipated [13, 14].

Some of the problems arise from the fact that IGF signaling is similar structurally and functionally to insulin signaling with shared intracellular signaling machinery and homologous receptor subunits, capable of forming hybrid receptors [15]. Structural similarity also poses a problem for the use of kinase inhibitors, which are likely to inhibit the kinase domain of both receptors [13]. In addition, cancer cells frequently express the A-isoform of the insulin receptor (IR), which lacks exon 11 (IR-A) [16]. IR-A has high affinity for both insulin and IGF-II, but is not targeted by IGF-IR blocking antibodies. Furthermore, the IGF-IR has widespread occurrence, and it is increasingly recognized that therapeutic, global manipulation of the receptor may have undesirable side effects related to metabolism, some of which had not been predicted prior to clinical trials [13].

A fundamental difference between insulin and IGF signaling is the mechanisms that control the level of bioactive ligand. In contrast to insulin signaling, IGF signaling is often based on local synthesis of either IGF-I or -II. The IGFs are present in tissues at relatively high concentrations, but at the same time, their ability to interact with IGF-IR is counteracted by differentially expressed IGF binding proteins, IGFBP-1 through -6. The high-affinity complexes of IGFBP and IGF constitute a local reservoir from which bioactive IGF can be released by different mechanisms, principally enzymatic cleavage of the IGFBP [17].

One such IGF-releasing enzyme is the metalloproteinase, pregnancy-associated plasma protein-A (PAPP-A), whose known substrates are IGFBP-4 [18] and -5 [19]. While several different proteinases are capable of cleaving IGFBP-5, physiological cleavage of IGFBP-4 may be limited to PAPP-A, suggesting that in the IGF system, PAPP-A and IGFBP-4 function as an interdependent pair [20, 21]. PAPP-A may therefore comprise a desirable therapeutic target to inhibit focal growth where an excessive level of bioactive IGF is caused by increased PAPP-A proteolytic activity. By using such a strategy, fewer side effects are likely because tissues where PAPP-A is absent will not be affected. To test the hypothesis that IGF-IR stimulation can be reduced by inhibition of PAPP-A, we generated a monoclonal antibody that selectively inhibits the cleavage of IGFBP-4 by targeting a unique substrate-binding exosite of PAPP-A, analyzed its efficacy *in vitro*, and assessed the principle *in vivo* by using a mouse xenograft model.

## RESULTS

### Targeting the proteolytic activity of PAPP-A towards IGFBP-4

**Figure 1:**
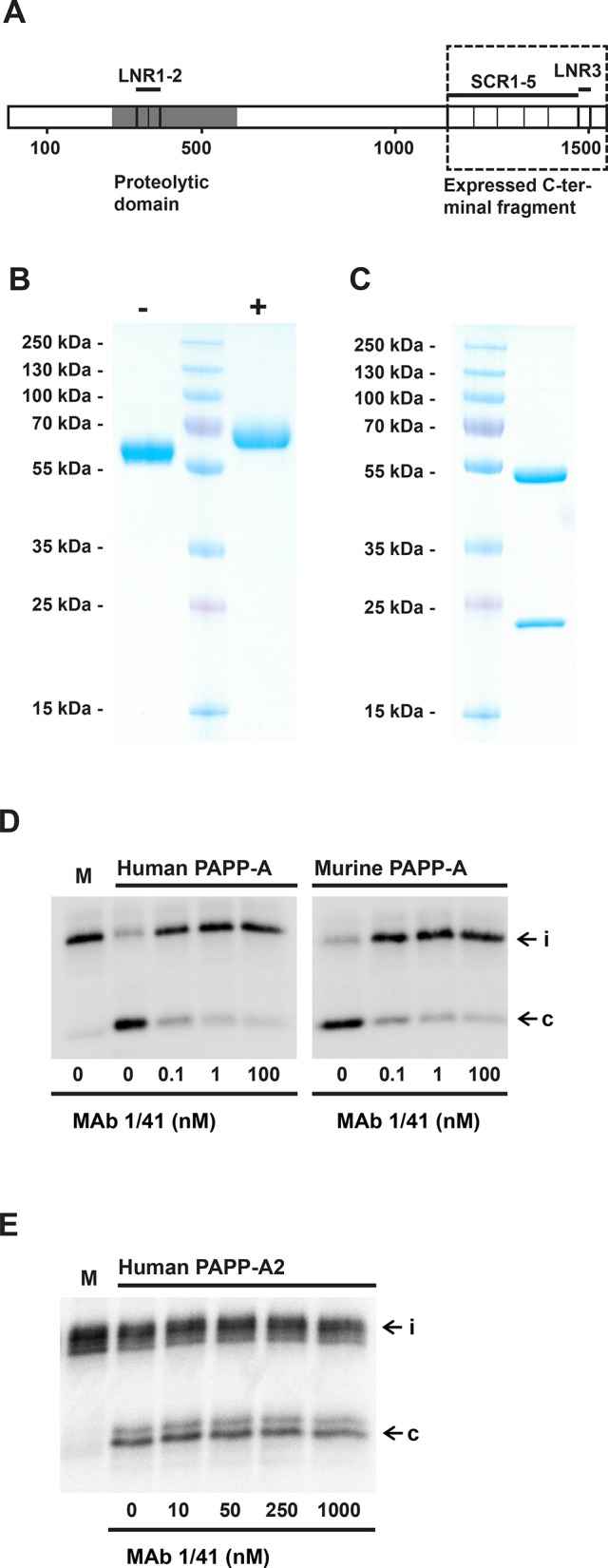
Development of an inhibitory antibody targeting the substrate-binding exosite of PAPP-A A, Schematic outline of the 200 kDa 1547-residue human PAPP-A subunit. Amino acid numbering, the positions of the three Lin12-Notch repeat modules (LNR1-3), five complement control protein modules (CCP1-5), and the proteolytic domain (grey) are shown. The position of the C-terminal recombinant fragment, used for selection of antibodies targeting the substrate-binding exosite located in the LNR3 region, is shown by the dashed rectangle. The LNR3 module is 100% conserved between mouse and man. B, Non-reducing (-) and reducing (+) SDS-PAGE of purified C-terminal fragment of human PAPP-A, PAPP-A(1133-1547), corresponding to the dashed rectangle of A. The molecular weights of marker proteins are indicated. C, Reducing SDS-PAGE of purified PAPP-A inhibitor, mAb 1/41, a murine IgG2a monoclonal antibody. The positions of molecular markers are indicated. D, Inhibition of cleavage of radiolabeled IGFBP-4 by recombinant human PAPP-A (200 pM, left panel) and recombinant murine PAPP-A (200 pM, right panel) by using mAb 1/41. Please note that in this experiment, C-terminally tagged IGFBP-4 was used, causing the N- and C-terminal cleavage fragments to co-migrate. Intact IGFBP-4 (i) and the co-migrating cleavage fragments (c) are indicated. E, MAb 1/41 does not inhibit cleavage of radiolabeled IGFBP-5 by recombinant human PAPP-A2 (100 pM). Concentrations of mAb 1/41 are specified.

**Figure 2:**
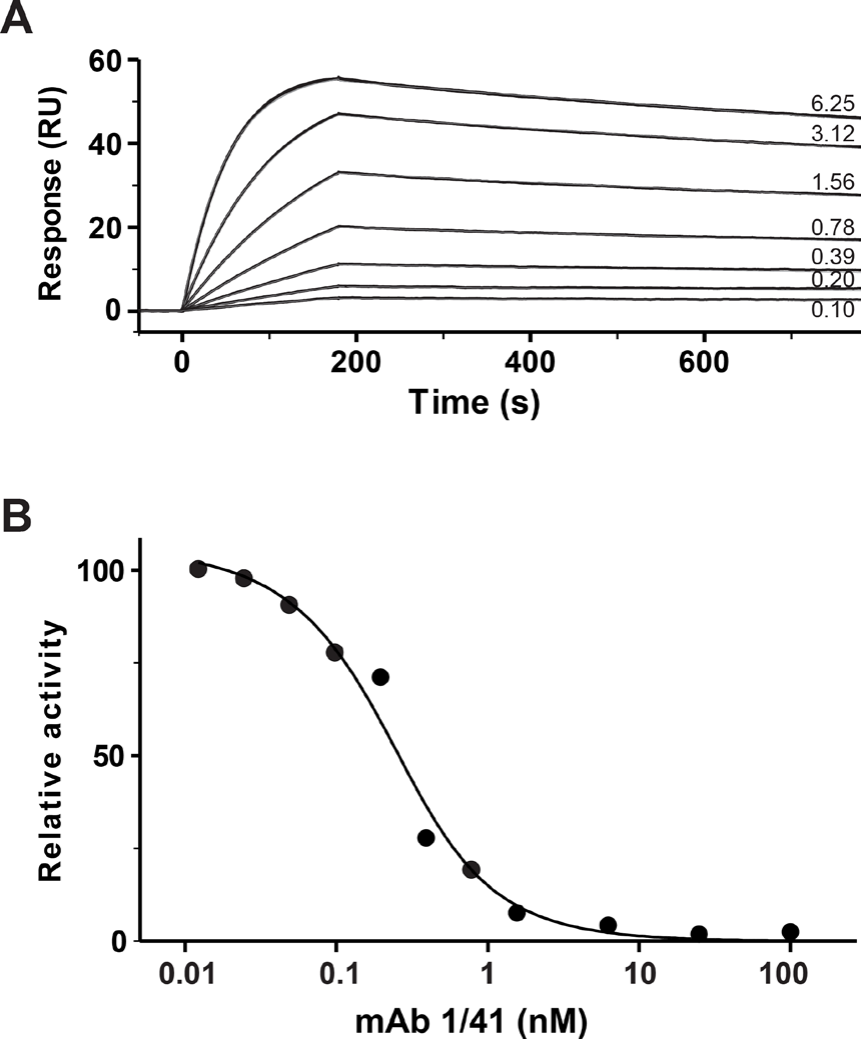
Characterization of inhibitory antibody mAb 1/41 A, MAb 1/41 binds PAPP-A with high affinity as demonstrated by surface plasmon resonance. Twofold serial dilutions of a human PAPP-A C-terminal fragment, PAPP-A(1133-1547), ranging from 6.25 nM to 97.5 pM, were injected over a surface with immobilized mAb 1/41. The experiment with 6.25 nM is shown in replicate, demonstrating good reproducibility. Recorded binding curves are shown in black, and a global 1:1 Langmuir fit is shown in grey. The fitted constants are: *k*_a_ = 3.168 ± 0.002 × 10^6^ M^−1^s^−1^, *k*_d_ = 3.080 ± 0.002 × 10^−4^ s^−1^, resulting in *K*_D_ = 9.72 × 10^−11^ M. B, Relative initial velocities of PAPP-A-mediated IGFBP-4 cleavage in the presence of increasing concentrations of mAb 1/41. Inhibitory constant, *K*_i_ = 1.35 ± 0.24 × 10^−10^ M. Using murine PAPP-A, mPAPP-A(1129-1545) [23], similar kinetics were obtained.

**Figure 3:**
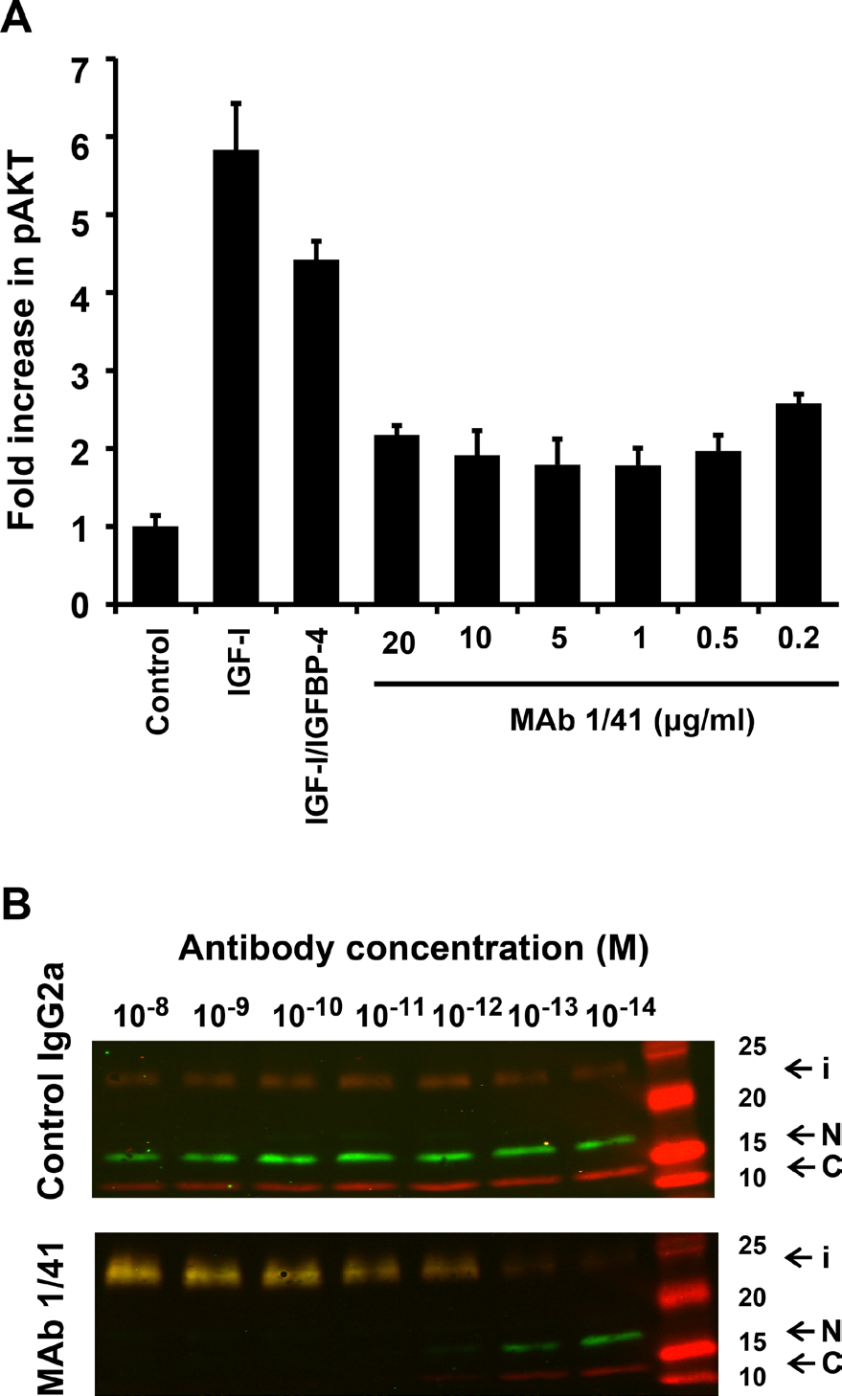
Inhibition of IGF-IR signaling and IGFBP-4 proteolysis by native PAPP-A in vitro A, Human lung carcinoma cells (A549) were cultured, and PAPP-A secreted into the culture medium was allowed to accumulate to a level of 48.6 ng/ml. The cells were treated with IGF-I (50 ng/ml), or IGF-I (50 ng/ml) first incubated with a fivefold molar excess of IGFBP-4 to allow formation of the inactive IGF/IGFBP-4 complex. Prior to treatment with the IGF/IGFBP-4 complex, the cells were incubated with varying doses of mAb 1/41 as indicated, and AKT phosphorylation was quantified. Average values +/− SDs of three independent experiments are shown. B, Forty-eight hour A549 conditioned medium was treated with varying concentrations of mAb 1/41 or a subtype control (IgG2a), as indicated. The ability of the treated culture medium to proteolyze IGFBP-4 was assessed by the addition of recombinant IGFBP-4 followed by Western blotting using antibodies specific for the N- and C-terminal regions of IGFBP-4. Intact IGFBP-4 (i) and N- (N) and C-terminal (C) cleavage fragments are indicated.

The C-terminally located LNR3 module of PAPP-A (Fig. 1A) harbors a unique substrate-binding exosite, which is required for binding and proteolytic cleavage of IGFBP-4 [22, 23]. To develop an inhibitory monoclonal antibody targeting this site, mice were immunized with full-length human PAPP-A. PAPP-A knockout mice [24] were used to ensure an efficient immune response towards conserved regions of the protein, in particular the LNR3 region which is highly conserved between species [25]. Antibodies secreted by hybridoma clones were screened successively for 1) recognition of the immunogen, 2) recognition of a recombinant C-terminal fragment of PAPP-A containing the target site (Fig. 1A and 1B), and 3) for lack of recognition of mutant PAPP-A(D1484A), in which the structure of LNR3 is disrupted [26]. Selected candidates were then screened for their ability to inhibit PAPP-A cleavage of IGFBP-4, and one antibody, mAb 1/41, was chosen for further characterization following production in milligram quantities. In reducing SDS-PAGE, this IgG2a antibody migrated as two distinct bands, suggesting homogenously glycosylation of its subunits (Fig. 1C). Qualitative analysis demonstrated that mAb 1/41 efficiently inhibited the cleavage of IGFBP-4 by both human and murine PAPP-A (Fig. 1D). Cleavage of IGFBP-5 by PAPP-A2 [27], the only other homologous proteinase (Fig. 1A), was not affected by mAb 1/41 (Fig. 1E), even at a large molar excess (10.000 fold) of mAb 1/41 over PAPP-A2. Analysis by surface plasmon resonance confirmed the suspected high-affinity binding of the antibody to the target site of recombinant PAPP-A (*K_D_* = 97 pM) (Fig. 2A), and by kinetic analysis, mAb 1/41 qualified as a potentially useful reagent for inhibition of PAPP-A activity with a favorable inhibitory constant (*K_i_*) of 135 pM (Fig. 2B).

### PAPP-A mAb 1/41 inhibits the activity of native PAPP-A and AKT phosphorylation in cell monolayers

Specific and IGF-dependent proteolytic cleavage of IGFBP-4 has previously been documented in conditioned media from non-small cell lung cancer cell lines [28], although the identity of the responsible proteinase was unknown then. Recently, the level of circulating PAPP-A was found to be increased in patients with lung cancer [29]. We therefore screened a panel of human lung cancer derived cells (Table 1), and picked the adenocarcinoma cell line, A549, as a model for PAPP-A-mediated stimulation of the IGF receptor based on its high level of PAPP-A secretion.

**Table 1:** Secretion of PAPP-A from selected human cancer cell lines Selected human lung cancer derived adenocarcinoma cell lines were cultured to confluency, and the levels of PAPP-A secreted into the culture medium were assessed by ELISA. ND, non-detectable.

Cell line	Level of PAPP-A in conditioned medium (ng/ml)
H1975	0.1
H358	0.1
HCC827	0.1
H1568	1
A549	164
Calu-3	ND
H1666	12

Using the A549 cell line, we assessed the ability of mAb 1/41 to inhibit IGF receptor signaling measured as inhibition of AKT phosphorylation in cultured monolayers of adherent cells (Fig. 3A). Compared to untreated cells, the addition of IGF-I caused a six-fold enhancement of AKT phosphorylation; a similar enhancement (four-fold) was observed with IGF-I pretreated with equimolar amounts of IGFBP-4. This might seem contradictory, considering the inhibitory action of IGFBP-4. However, IGF-I is released from the inactive IGF/IGFBP-4 complex by the high levels of PAPP-A secreted by the A549 cells. In agreement with this, we observed a dramatic decrease in AKT phosphorylation when the cells treated with the IGF/IGFBP-4 complex were also treated with inhibitory mAb 1/41. The effect appeared to be saturable and to decrease with lower doses of mAb 1/41.

To verify at the level of proteolysis that mAb 1/41 was capable of inhibiting the native PAPP-A secreted from the A549 cells, culture medium from these cells was treated with a variable amount of mAb 1/41, or a control antibody of the same subtype. The control antibody had no effect on the proteolytic activity towards IGFBP-4, while mAb 1/41 efficiently inhibited cleavage (Fig. 3B). This experiment demonstrates that not only recombinant PAPP-A (Fig. 1), but also native PAPP-A can be targeted by mAb 1/41.

### Assessment of the ability of mAb 1/41 to inhibit PAPP-A activity in vivo

**Figure 4:**
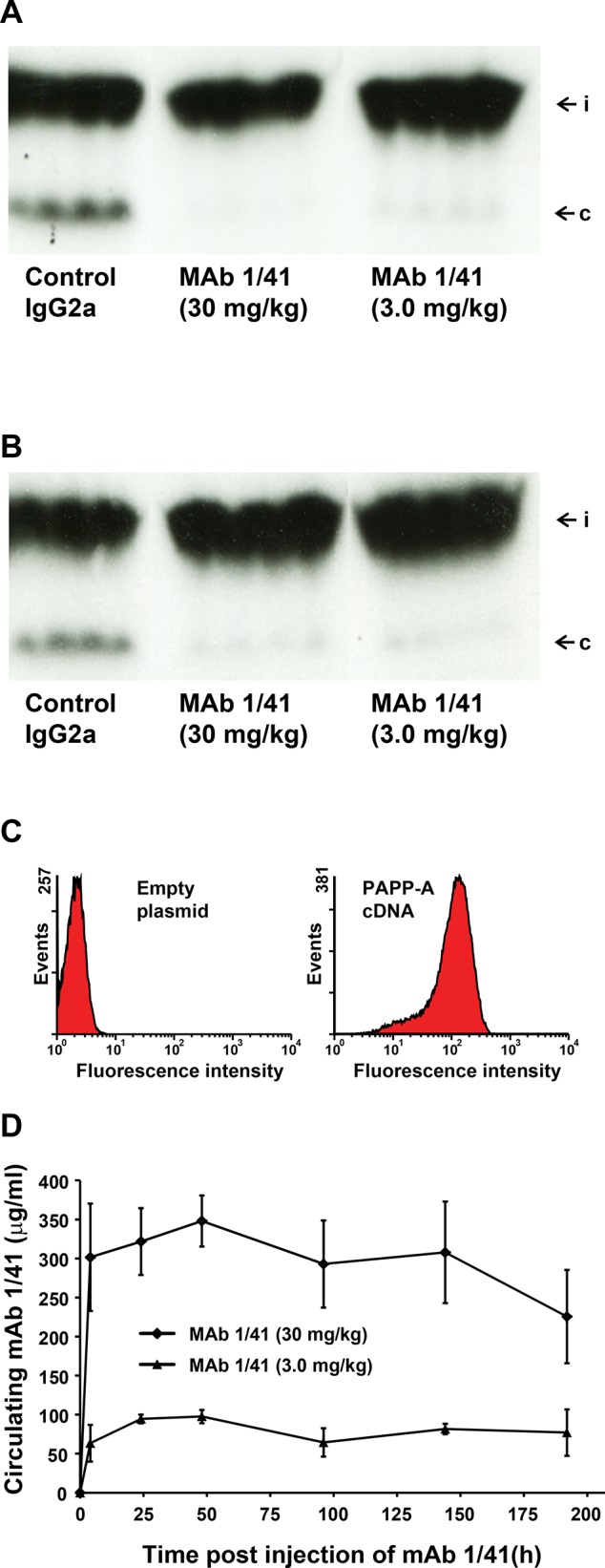
Inhibition of PAPP-A-mediated IGFBP-4 proteolysis in vivo A, Adult, male mice were delivered IgG2a (30 mg/kg) or inhibitory mAb 1/41 (30 or 3.0 mg/kg) by intraperitoneal injection. Proteolysis in the circulation of exogenously administered, radiolabeled IGFBP-4 was assessed by gel electrophoresis and autoradiography 24 hours following antibody delivery. Individual lanes are results from individual mice. The positions of intact (i) and co-migrating cleavage fragments (c) are indicated. To allow proteolysis of IGFBP-4 in the circulation [30, 31], injection of heparin to release surface bound PAPP-A was given prior to the injection of IGFBP-4. B, A similar experiment was carried out 8 days after delivery of the antibody. C, Flow cytometry demonstrating strong binding of mAb 1/41 to cells transfected with PAPP-A cDNA (right panel) but not to cells transfected with empty plasmid cDNA (left panel). D, Example showing circulating levels of mAb 1/41 measured at various times after intraperitoneal administration of 30 or 3.0 mg/kg.

To address the question whether mAb 1/41 is a functional inhibitor of PAPP-A *in vivo*, mice were administered the inhibitor or a control antibody intraperitoneally. Four hours later, the mice were given heparin to increase the concentration of PAPP-A present in the circulation [30, 31], and trace amounts of radiolabeled IGFBP-4 pre-bound with IGF-II was injected into the tail vein. Proteolytic cleavage of the injected IGFBP-4 was then assessed by autoradiography of serum samples separated by SDS-PAGE (Fig. 4A). No cleavage of IGFBP-4 was observed at the high dose of mAb 1/41. At a lower dose, cleavage was not completely inhibited. Eight days after injection of the antibody, faint traces of IGFBP-4 cleavage fragments were present at both the high and the low dose of antibody, although efficient inhibition was still evident (Fig. 4B). We therefore conclude that mAb 1/41 is capable of inhibiting PAPP-A present in the extracellular environment of a living organism. By using flow cytometry, we further demonstrated that mAb 1/41 is capable of binding the fraction of PAPP-A that *in vivo* is likely to be bound to surfaces of cells [30] (Fig. 4C).

Finally, we assessed the pharmacokinetic properties of mAb 1/41 in mice (Fig. 4D). A high (30 mg/kg) and a low (3.0 mg/kg) dose of the antibody were injected intraperitoneally, and the circulating levels were monitored. For both the high and the low dose, the level of antibody had decreased to about 65% of the initial concentration following eight days.

### PAPP-A mAb 1/41 inhibits growth in a xenograft model

**Figure 5:**
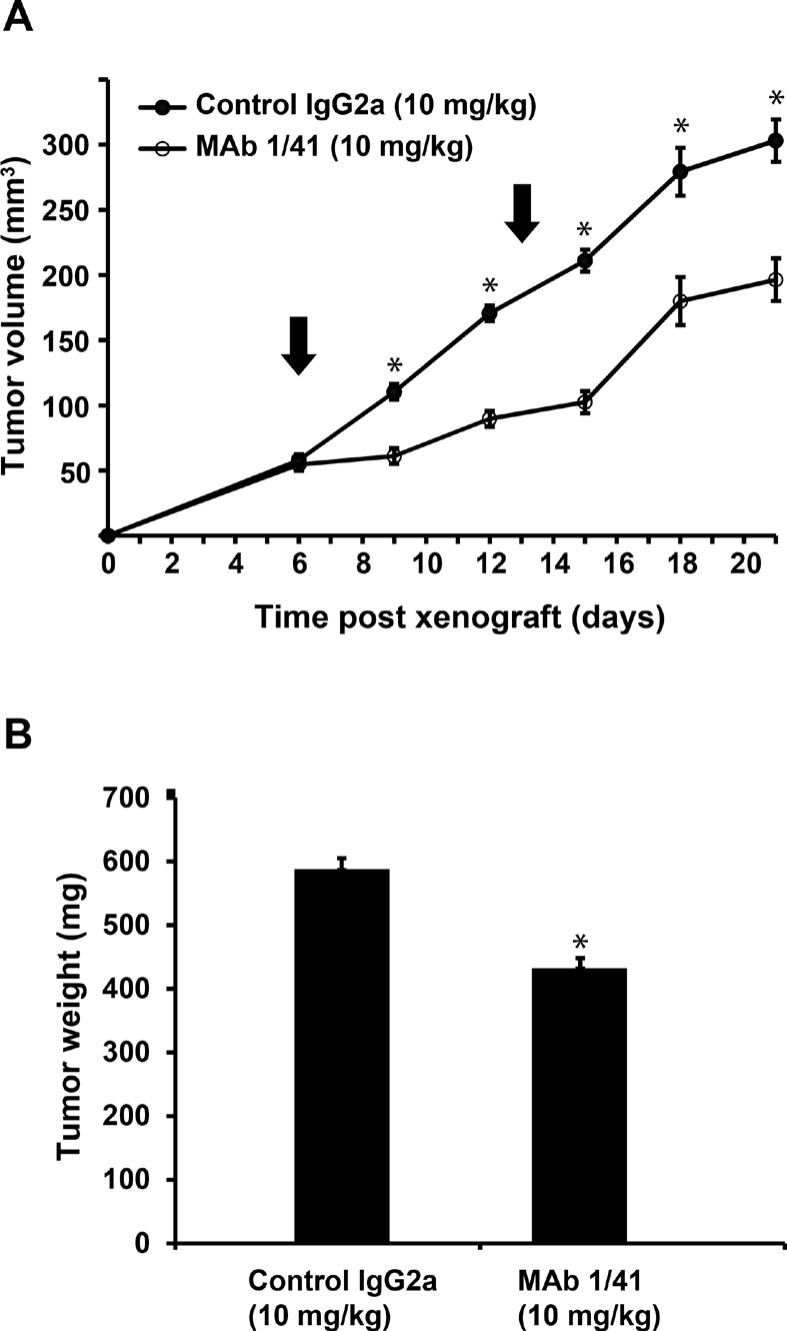
Inhibition of xenograft tumor growth using PAPP-A inhibitor mAb 1/41 A, Athymic nude mice were delivered A549 cells subcutaneously and treated at day 6 and 13 with IgG2a or mAb 1/41 at doses of 10 mg/kg. Each group contained 15 mice. Tumor volumes were assessed and average values +/− SEMs were plotted as a function of time post-xenografting. B, Average tumor masses following three weeks for mice treated with control IgG2a and mAb 1/41. *p < 0.001.

Based on the above, xenograft experiments were carried out to determine the effectiveness of targeting PAPP-A *in vivo*. A549 lung cancer cells were implanted subcutaneously in immunocompromised mice, and on day 6 and 13, the mice were treated with 10 mg/kg of mAb 1/41, or an subtype control antibody. Following onset of treatment, the tumor sizes were measured twice weekly for two weeks. The animals were then sacrificed and the tumor masses were determined by weighing. Treatment with mAb 1/41 showed a significant reduction in growth rate (Fig. 5A), most prominent until day 15, where the total reduction was greater than 50%. The difference in tumor mass at harvest (day 21) was significant between the two groups (Fig. 5B).

**Figure 6:**
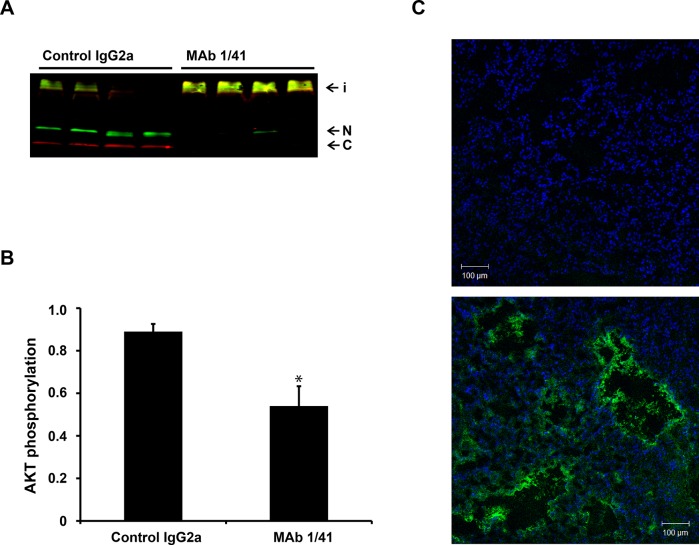
Intra-xenograft inhibition of PAPP-A activity following treatment with inhibitory monoclonal antibody A, A549 xenografts from mice treated with IgG2a or mAb 1/41 were homogenized and the ability of the homogenate to proteolyze IGFBP-4 was assessed by the addition of recombinant IGFBP-4 followed by Western blotting using antibodies specific for the N- and C-terminal regions of IGFBP-4. Intact IGFBP-4 (i) and N- (N) and C-terminal (C) cleavage fragments are indicated. B, Quantification of AKT phosphorylation in xenograft tissue following treatment with IgG2a control or mAb 1/41. *p < 0.01. C, Immunofluorescent detection of mAb 1/41 penetrance within the xenograft was conducted using a FITC-labeled, anti-mouse IgG2a antibody (green) in untreated (upper panel) and mAb 1/41 treated (lower panel) mice.

The ability of tumor extracts to proteolyze IGFBP-4 was then assessed. As expected, extracts from mice treated with the inhibitory antibody showed a clear reduction in proteolysis (Fig. 6A). Concordantly, we found a significant reduction in IGF signaling, as measured by AKT phosphorylation, between the groups (Fig. 6B). Finally, we verified by IgG2a immunostaining that the antibody was present in the xenograft tissue at the end of the experiment (Fig. 6C). Although, based on this experiment, it is not possible to determine the degree of mAb 1/41 penetration, the presence of the antibody within the tissue is evident and most likely the reason for the reduced IGF signaling.

## DISCUSSION

Despite the enormous interest in therapeutic manipulation of IGF signaling, very few efforts have been made to target upstream regulatory molecules in the extracellular part of the IGF signaling network. Use of IGFBPs to sequester IGF bioactivity is being attempted [15], but there are no prior reports in which the proteolytic activity of an IGFBP proteinase has been targeted. Reasons for that might be that some proteinases shown to cleave IGFBPs, e.g. matrix metallloproteinases, are known to be involved in proteolytic regulation outside the IGF system [17]. Hence, proteolytic targeting clearly can have unwanted and unpredictable consequences. For two reasons we believe that targeting of PAPP-A is different: First, targeting the substrate-binding exosite of PAPP-A selectively inhibits cleavage of IGFBP-4 [22, 23]. Second, IGFBP-4 is cleaved in an unusual IGF-dependent manner by PAPP-A [19], and does not appear to be a physiological substrate of other proteinases [20, 21].

By immunizing PAPP-A knockout mice, we have developed a prototype PAPP-A inhibitor in the form of a monoclonal antibody, mAb 1/41, that targets the substrate-binding exosite, present in the highly conserved C-terminal LNR module (LNR3) of PAPP-A. The antibody binds PAPP-A with picomolar affinity (*K_D_* = 97 pM), is specific for PAPP-A, and shows excellent inhibitory kinetics (*K_i_* of = 135 pM) towards the cleavage of IGFBP-4 for both human and murine PAPP-A (Fig. 1 and 2).

Using cultured A549 cells, which secrete both PAPP-A and IGFBP-4, we demonstrated that mAb 1/41 is capable of inhibiting both proteolytic cleavage of IGFBP-4 and IGF signaling, the latter assessed by measuring decreased AKT phosphorylation (Fig. 3). This experiment provides an *in vitro* model system in which the coupled action of IGFBP-4 and PAPP-A regulates IGF receptor stimulation. It is important to note that the inhibitory antibody is effective towards native PAPP-A, not only recombinant PAPP-A, which was used in the selection process of the antibody. We further demonstrated that mAb 1/41 is capable of exerting its inhibitory function *in vivo* towards native murine PAPP-A (Fig. 4).

Cleavage of IGFBP-4 and resulting release of bioactive IGF in close proximity to the IGF-IR is believed to be important for efficient IGF-IR stimulation [20], in agreement with the ability of PAPP-A to bind to cellular surfaces [30]. Because the cell surface binding site of PAPP-A is located in modules SCR3 and SCR4 (Fig. 1A), close to the LNR3 module containing the epitope of mAb 1/41, the accessibility of the epitope of surface-bound PAPP-A might be compromised. However, the simultaneous binding of PAPP-A to the cell surface and to mAb 1/41 demonstrates that this is not the case (Fig. 4C). Therefore, mAb 1/41 can inhibit PAPP-A present in tissues, where it is reasonable to assume that the majority of PAPP-A is associated with cell surfaces.

To then test the hypothesis that targeting of PAPP-A can indirectly reduce excessive IGF-IR stimulation *in vivo*, we assessed the ability of mAb 1/41 to inhibit growth in a xenograft mouse model based on subcutaneously implanted A549 cells (Fig. 5). We found, in agreement with our hypothesis, that inhibition of PAPP-A decreased the tumor growth rate, even in this experimental system with secretion from A549 cells being extremely high compared to measured physiological levels of the protein. Our results therefore provide proof of principle for a strategy of targeting upstream extracellular components of the IGF system *in vivo* to reduce IGF signaling locally. For further studies, it may be desirable to substitute the current heterotopic xenograft model with a more sophisticated model system, in which extracellular IGF system components are characterized, and dose and treatment regimen can be optimized.

The involvement of PAPP-A in the development or growth of different types of cancer is increasingly suggested in the literature. These include ovarian cancer, renal cancer, mammary cancer, gastric cancer, lung cancer, and pleural mesothelioma [29, 32-41]. Furthermore, studies in mice have shown that *PAPP-A* transcription is activated by mutant p53 [42], and, of particular interest, *PAPP-A* null mice have a markedly reduced incidence of spontaneous cancers of age [43]. Finally, recent reports demonstrate that a shRNA- [39] or siRNA-mediated [41] reduction in *PAPP-A* gene expression causes reduced tumor cell proliferation in xenograft models.

It is therefore reasonable to hypothesize that in some cancers, where the tumor phenotype is driven by increased IGF signaling, increased PAPP-A activity may be part of the underlying mechanism. In these cases inhibition of PAPP-A might be beneficial – and include the advantage of local and indirect targeting of the IGF-IR, rather than direct global targeting which lacks specificity and is likely to interfere with metabolism. Importantly in this regard, the PAPP-A knockout mice do not show secondary endocrine alterations [24].

Furthermore, PAPP-A may serve as a predictive biomarker to identify probable responders, the lack of which is currently recognized as a major problem in cancer treatment based on IGF-IR antagonists [44]. In line with this, it is also possible that IGFBP-4 cleavage fragments, generated by PAPP-A activity, have biomarker value, either for patient selection or as a tool to monitor efficacy of the treatment.

In conclusion, we have developed the concept of indirect inhibition of IGF-IR signaling by inhibiting a proteolytic enzyme, PAPP-A. Inhibition of PAPP-A prevents the generation of bioactive IGF by specific cleavage of IGFBP-4, and we have demonstrated that this local targeting approach works *in vivo*. The advantage of targeting PAPP-A is principally increased specificity, fewer side effects, as well as its potential use as a biomarker. Future work will aim at optimizing the efficacy of PAPP-A inhibition in more translational model systems, and to assess the possible value of combining PAPP-A inhibition with other therapeutic strategies.

## METHODS

### Expression and purification of recombinant protein

Human embryonic kidney 293T cells (293tsA1609neo) were cultured in high glucose Dulbecco's modified Eagle's medium (DMEM) supplemented with 10% fetal bovine serum (FBS), 2mM glutamine, nonessential amino acids, and gentamicin (Invitrogen). Cells were plated onto 6-cm tissue culture dishes and transiently transfected 18 h later by calcium phosphate co-precipitation using 10 μg of plasmid DNA. The plasmids encoded the following: Wild-type human PAPP-A [45], and mutants E483Q [45] and D1484A [26], wild-type murine PAPP-A [46], human c-Myc-His tagged IGFBP-4 [47], or a c-Myc-His tagged version of plasmid PA(1133–1547) [22], encoding a C-terminal fragment of human PAPP-A, starting at SCR module 1, PAPP-A(1133-1547). After 48 h, the culture media were harvested and cleared by centrifugation, or the cells were further cultured in serum free medium, CD293 (Invitrogen), to facilitate purification. Tagged proteins were purified by nickel affinity chromatography using Chelating-Sepharose Fast Flow beads (1 ml) (Amersham Biosciences). The column was washed with 1 M NaCl, 50 mM sodium phosphate, pH 5.5, and bound protein was eluted with phosphate-buffered saline containing 20 mM EDTA.

### Generation of inhibitory antibody

PAPP-A knockout mice [24] were immunized with human PAPP-A complex purified from human pregnancy serum [48]. Initial injections of 50 μg were given subcutaneously in Freund's Complete Adjuvant (MP Biomedical, 0855828). After one month, each mouse was given a booster injection in Freund's Incomplete Adjuvant (MP Biomedical, 0855829). Based on screening of tail blood on plates coated with the antigen, good responders were selected for final boosting following one additional month: For four consecutive days, mice received approximately 160 μg of the antigen intraperitoneally. On the fifth day, the mice were sacrificed and the spleens removed aseptically. Spleen cells were centrifuged, aliquoted and stored in liquid nitrogen. Fusion with SP2/0 cells was performed with one aliquot of the spleen cells. Culture supernatants of picked clones were screened on antigen coated plates, and positive clones were re-cloned. The clones were then screened for recognition of purified human C-terminal PAPP-A fragment, PAPP-A(1133-1547), coated onto plastic, and for lack of recognition of PAPP-A mutant D1484A. The latter PAPP-A mutant was immobilized to the wells by using polyclonal rabbit anti-PAPP-A [48]. Selected clones were cultured for antibody production and purification on protein A Sepharose (GE Healthcare). These clones were finally screened for their ability to inhibit PAPP-A (human and murine) cleavage of IGFBP-4 (see above). A kit (IsoQuick Strips, Sigma Aldrich, cat. no. I9285) was used for isotyping. Sufficient amounts of the mAb 1/41 antibody was produced and purified by a commercial service provider (BioServ UK Ltd., Sheffield, UK). Prior to use in animal experiments, the biochemical properties of the antibody was verified by the methods described above.

### Analysis of IGFBP cleavage in vitro

To assess proteolytic inhibition, the proteolytic activity of PAPP-A against IGFBP-4 was analyzed as previously described in detail [49]. Briefly, purified IGFBP-4 was labeled with 125I (Amersham Biosciences), and cleavage reactions (t = 60 min) were carried out in 50 mM Tris-HCl, 100 mM NaCl, 1 mM CaCl2, pH 7.5, in the presence of a molar excess of IGF-II (Diagnostic Systems Laboratories) at 37 °C. Concentrations were: PAPP-A, 200 pM; IGFBP-4, 10 nM; IGF-II, 100 nM. In some reactions, purified antibody was added in known amounts as specified. Reactions were terminated by the addition of SDS-PAGE sample buffer supplemented with 25 mM EGTA. For kinetic analysis, reaction times were 0, 30, 60, and 120 min. Cleavage products were separated by 10-20% SDS-PAGE and visualized by autoradiography. The degree of cleavage was determined by quantification of band intensities using a Typhoon imaging system (GE Healthcare), and background levels (mock signals) were subtracted. Relative initial velocities (V_i_/V_0_) were determined by linear regression assuming no substrate depletion. Quantitative analyses for inhibitory constant (*K*_i_) determination were carried out with GraphPad Prism 5.0 software using the Morrison *K*_i_ model (competitive inhibition). For semi-quantitative experiments, unlabeled IGFBP-4 (R&D Systems) was used, and proteolytic activity was assessed by the separation of cleavage products in Western blotting. Briefly, samples were separated by 4-20% SDS-PAGE, blotted onto a PVDF membrane (Millipore), blocked with LI-COR blocking buffer, and probed for the N- and C-terminal fragment of IGFBP-4 using specific antibodies (ab92625 and ab153654, Abcam) by incubation for 20 h at 4°C. Fluorescently labeled secondary antibodies (LI-COR) were used for detection of intact and cleaved IGFBP-4, images were captured using a LI-COR Odyssey scanner, and intensities measured using the Image J software. Cleavage (t = 30 min at 37 °C) of IGFBP-5 by human PAPP-A2 was carried out as previously detailed [27], using 10 nM IGFBP-5, 100 nM IGF-II, 100 pM PAPP-A2 and a variable concentration (0-1000 nM) of mAb 1/41.

### Surface plasmon resonance analysis

Surface plasmon resonance experiments were carried out on a Biacore T200 (GE Healthcare). Purified antibody was captured in flow cell 2 of a protein A coupled PAD 500L chip (XanTec Bioanalytics) to a density of 100 response units. To collect kinetic binding data, a twofold serial dilution of the analyte, PAPP-A(1133-1547), ranging from 6.25 nM to 97.5 pM, in 10 mM HEPES pH 7.4, 500 mM NaCl, 1 mM CaCl2 and 0.05% Tween-20, was injected over flow cells 1 and 2 at 30 μl/min. The association phase was 180 s, followed by a 600 s dissociation phase. Binding analysis was performed at 25 °C. At the end of each binding cycle, all surfaces were regenerated by a 60 s injection of 10 mM glycine pH 1.7. Analyte concentrations were determined by amino acid analysis. Data were collected at a rate of 10 Hz. Recorded signals were subtracted the background signal, as determined by the response obtained from a reference cell (flow cell 1) without antibody capture. Global fitting of a 1:1 Langmuir model was performed, using the Biacore T200 Evaluation Software, version 1.0

### Determination of AKT phosphorylation

A panel of human lung cancer derived cell lines (ATCC, Table 1) were assessed by ELISA for the presence of PAPP-A in the culture medium. Human alveolar adenocarcinoma cells (A549, ATCC) [50] were selected and thereafter maintained in DMEM containing 10% FBS, 2 mM glutamine, penicillin and streptomycin. The cells were allowed to reach confluence in a 96-well plate, washed three times and switched to DMEM containing antibiotics and 0.1% bovine serum albumin for 16 hours to allow PAPP-A secretion and accumulation in the medium. Varying doses of an irrelevant control IgG2a antibody (BioXCell) or PAPP-A mAb 1/41 were added 1 h prior to the addition of recombinant human IGF-I (50 ng/ml, R&D Systems), or IGF-I and human recombinant IGFBP-4 (50/500 ng/ml, R&D Systems) pre-incubated for 30 minutes to allow the IGF/IGFBP-4 complex to form. Fifteen minutes after IGF stimulation, conditioned media were collected and cells fixed and permeabilized for In-Cell Western analysis (LI-COR). An anti-phospho-AKT antibody (Novus Biologicals) was used to assess IGF receptor signaling using the LI-COR Odyssey system. Levels of PAPP-A were determined by ELISA (AL-101, Ansh Labs).

### Flow cytometry

Transfected 293T cells were detached with 20 mM HEPES, 150 mM NaCl, pH 7.5 containing 5mM EDTA 48 h post-transfection with PAPP-A cDNA and washed with cold culture medium containing 5% FBS. The cells were incubated on ice with primary antibody (mAb 1/41) diluted in culture medium with 10% FBS to 0.5 μg/ml for 1 h. After washing three times, the cells were incubated with Alexa Fluor 488 goat anti-mouse diluted to 2 μg/ml for 1 h. After 3 washes, the cells were suspended in PBS with 2% paraformaldehyde and analyzed on a Beckman Coulter FC500 instrument. At least 10,000 cells were analyzed.

### Analysis of IGFBP-4 cleavage *in vivo*

Adult, male FVB mice (Charles River Laboratories) were administered control IgG2a (BioXCell) or PAPP-A mAb 1/41 by intra-peritoneal injection of 3.0 or 30 mg/kg. Serum was collected from previously antibody-treated mice by eye bleed at various time points for the determination of circulating levels of PAPP-A mAb 1/41 (eBioscience). At 24 h and 8 days post injection of antibody, the inhibitory potential in the circulation was assessed: Mice were administered 10 U heparin (Sigma) (to release cell surface-associated PAPP-A) followed by radiolabeled IGFBP-4 (cf. above) and IGF-II via tail vein injection. Animals were sacrificed 10 minutes after the injection of trace amounts of 125I-labeled IGFBP-4. Serum was collected and stored at -80 prior to separation by 4-20% SDS-PAGE. The gels were dried and exposed to X-ray films (Kodak) for visualization of IGFBP-4 proteolysis.

### Xenograft model

Adult, female athymic nude mice (Charles River Laboratories) were allowed to acclimatize for one week following delivery to the animal facility. The mice were housed in pathogen-free conditions for the duration of the study. A549 cells were propagated, lifted by trypsinization, washed three times with phosphate-buffered saline (PBS), and counted. Animals (n = 15 per group) were delivered 5.0 x 10^5^ cells suspended in a volume of 100 μl PBS subcutaneously mid-thigh. The mice were followed for six days, and tumor volumes (in mm^2^) were determined by two-dimensional caliper measurement using the modified ellipsoid formula, V_t_ = ½ (L x W^2^), where L = length in mm, and W = width in mm, and randomized to treatment group. Treatments were initiated at day 6 by intraperitoneal injections of control IgG2a or PAPP-A mAb 1/41 at 10 mg/kg. A second dose was given one week later for a total of two treatments. Tumor volumes were determined every three days. The animals were sacrificed three weeks after xenografting, the tumors were weighed, and serum was collected. Portions of the tumor were flash-frozen for biochemical analysis or OCT-embedded for immunohistochemistry and stored at -80 until further analysis. The levels of AKT phosphorylation in tumor extracts from mice treated with mAb 1/41 (n = 6) or control IgG2a (n = 5) were assessed as above. Tumor extracts were also assessed for the ability to cleave IGFBP-4. All protocols involving animals were reviewed and approved by the Institutional Animal Care and Use Committee of Mayo Clinic.

### Immunohistochemistry

Cryosections (5 μm) of xenograft tumor were fixed in methanol and dried on glass slides. Sections were rehydrated in PBS, blocked in protein-free buffer (DAKO), and murine IgG2a was detected using a FITC-labeled, anti-mouse IgG2a antibody (Jackson ImmunoResearch). Images were captured using a Zeiss fluorescent microscope.

### Statistical analysis

Results are expressed as average values +/− SDs for the indicated number of experiments. Statistical analyses were performed using ANOVA, followed by multiple comparisons.
